# Qualitative Research on the Primary Effect of Fish Pet Ownership Using the Bottleium, a Bottle-Type Aquarium, on Community-Dwelling Older Adults in Japan: A Potential Preventive Measure towards Social Isolation

**DOI:** 10.3390/geriatrics6010017

**Published:** 2021-02-10

**Authors:** Mai Takase, Ryogo Ogino, Keishiro Yoshida, Hikari Kusu, Tetsuya Kenmochi, Jun Goto

**Affiliations:** 1 The Institute of Gerontology, University of Tokyo, Tokyo 113-8656, Japan; ryogoogi@cc.saga-u.ac.jp (R.O.); goto.jun.w@tokai.ac.jp (J.G.); 2 Graduate School of Teacher Education, Saga University, Saga 840-8502, Japan; 3 Gex Corporation, Osaka 578-0903, Japan; yoshida@gex-fp.co.jp (K.Y.); hikari-k@gex-fp.co.jp (H.K.); kenmochi@gex-fp.co.jp (T.K.); 4 School of Engineering, Tokai University, Tokyo 151-8677, Japan

**Keywords:** pet ownership, ornamental fish, community-dwelling older adults, social isolation, *ikigai-kan*

## Abstract

Aging increases the risk of social isolation, which could lead to conditions such as depressive mood. Pet ownership is known to reduce social isolation. However, previous studies have mainly focused on mammals as pets, which could be difficult at old age. A small ornamental fish is relatively easy to culture and might be a suitable alternative. In this research, we aimed to elucidate the possible effects of fish ownership on the psychological state of community-dwelling older adults in Japan. A Bottleium, a bottle-type aquarium, was selected to lower the burden of fish ownership. A workshop was hosted in 2019 and participants brought home their own Bottleium, with fish and water snail inside. Nineteen participants gave consent to the follow-up interview a month later. Five themes, “observation of fish and water snail”, “interaction between the fish and the owner”, “taking care of the fish as pet owner”, “facilitation of interpersonal interaction”, and “development of support system”, emerged from thematic analysis. The promotion of animal-to-human, and human-to-human interaction and development of responsibility could relate to a sense of social inclusion and *ikigai-kan*, a purpose of life. Fish ownership, when using equipment that suits the physical capability of older adults, could act as a positive stimulus.

## 1. Introduction

Japan is one of the leading countries with an aging society; a high percentage of the population is over the age of 65 [1]. Older adults face physical decline and tend to have lower motivation in participating in social activities [2], increasing the risk of social isolation and loneliness [3]. This can lead to serious symptoms such as depressive mood [4]. Pet ownership leads to a lower level of loneliness [5] and is one of the effective preventive measures. Other studies have demonstrated that having a pet as a companion animal has a protective effect against loneliness [6] and loss of social connection [7,8]. Furthermore, having a pet can be critical for the health and psychological well-being of older adults, since pets provide adults with company at home and, in some ways, require constant attention.

Previous studies about the effect of pets on older people have reported about mammalian species, reptiles, birds, and fish as pets, and mammals in particular have been extensively studied [7,9,10,11]. Applebaum (2020) reported that younger-aged people, before the age of 60, have the highest percentage of mammalian pet ownership [12]. Other studies report that compared to the younger generation, the percentage of people with pets are low in generation above the age of 60 [13]. Despite this condition, some studies focused on pet ownership in geriatric years. For example, a study by Taniguchi et al. compared the level of frailty between older adults who were never dog/cat owners with current and past owners. The latter had lower odds of developing frailty [10,11]. These findings present promising results of pet ownership; however, the physical decline associated with ageing and the life span of dogs and cats may prevent older adults from newly welcoming dogs or cats as pets. Meanwhile, various types of domestic ornamental fish require less care and have a shorter life span of two to three years. Statistics show that the number of people who own domestic fish is less likely to change compared to those who own dogs or cats [6]. Furthermore, a finding by Langfield and James suggested beneficial effects of fish ownership in mixed aged groups in Australia [14]. The conventional studies report the benefits of pet ownership including fish. On the other hand, research studies focusing on fish pet ownership and older adults are scarce. Some positive implications are reported regarding the effect of aquarium observation on the health of residents at specialized dementia units [15,16]. However, the action of residents is limited to fish observation and not pet ownership. Therefore, future research required is qualitative study which elucidates the effecting process of fish ownership towards independently living community-dwelling older adults.

**Figure 1:**
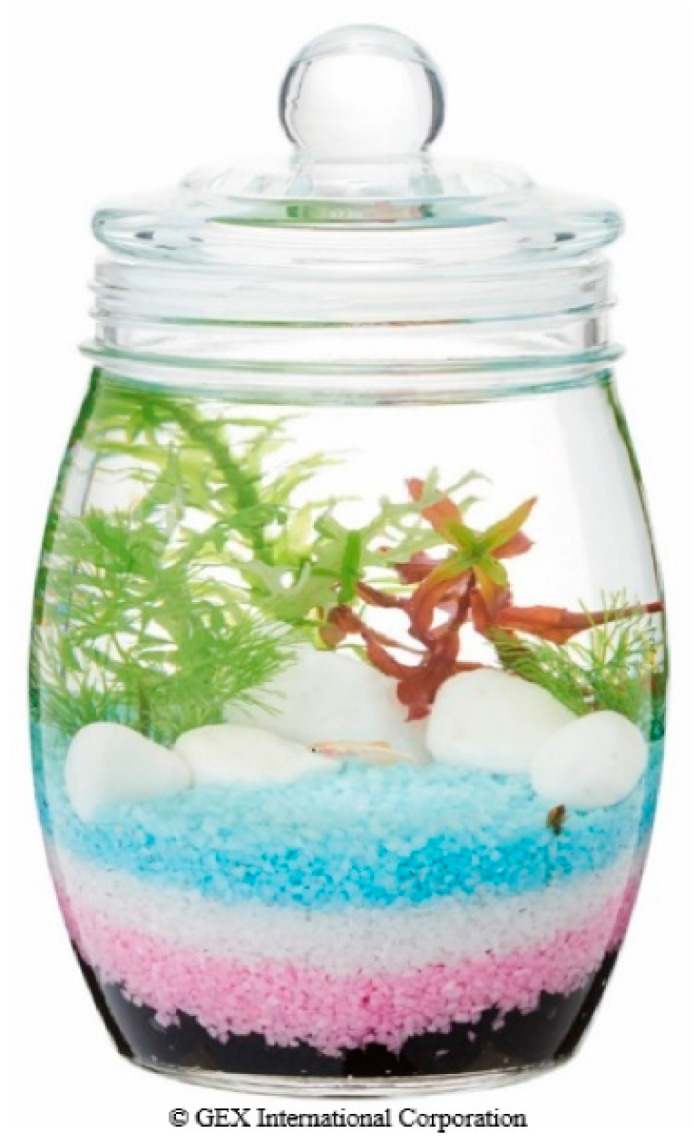
An Example of a Bottleium. One fish and one water snail are allowed in each bottle. An aeration and filtering system is not required.

Fish might be an alternative to mammalian pet ownership if provided with suitable culturing equipment. The conventional study was conducted with fully equipped domestic fish tanks [14]. Maintaining the filtering system and changing large volumes of water can eventually become difficult for older adults. To solve this problem, a novel type of aquarium named “Bottleium”, a neologism using the term bottle and aquarium, was selected (Figure 1). The Bottleium uses a small-size glass bottle that one can easily move. It does not require filtering or an aeration system. One fish and one water snail are allowed in each bottle and oxygen to the animals is provided by the water plant planted in the center of the bottle. Fishes cultured in the Bottleium live for few years, which is equivalent to their original life span. The size and ease of maintaining the Bottleium could enable ownership by older adults.

In this study, we used the Bottleium as a way to own a pet fish. First, difficulty of maintaining and handling the Bottleium was questioned as basic information. Then, the effect of ornamental fish ownership was studied qualitatively with the aim to extract factors that may be associated with the psychological state of older adults.

## 2. Materials and Methods

### 2.1. Ethical Approval

The overall structure of the research was explained at the beginning of the workshop and participants were asked to write their names and address down if they gave consent to be a part of the research. This research was approved by the Life Science Ethics Committee of the University of Tokyo.

### 2.2. Participants

A workshop about Bottleium was hosted in the community space of T housing complex (Chiba prefecture, Japan) in 2019. The community space is usually used to host events targeting community-dwelling citizens residing in the nearby areas. The notice of the Bottleium workshop was posted at the community space and around the housing complex for approximately one month prior to the actual date. Due to the original rule of community space operation, no previous registration to the workshop was allowed. The events hosted at the space have to be open to everyone who is interested in the activity. The participants came to the community space and joined the workshop on a first-come, first-served basis. The duration of the workshop was approximately two hours. Each participant built their own Bottleium (11 cm × 11 cm × 20 cm) by putting soil and color sand into the bottle, planting a water plant, and filling the bottle with water (Figure 1). The instructor taught participants the water changing method and at the end of the workshop, a small ornamental fish (about 3 to 4 cm) and water snail were added to the bottle. Fish food was provided to each participant and the participants brought home their own Bottleium.

### 2.3. Interview Data Collection and Analysis

Semi-structured interviews were conducted with the 19 workshop participants who gave consent. The interviews were conducted one month after the workshop. Participants were questioned using an interview guide that included open-ended questions regarding difficulties in owning or maintaining the Bottleium, and changes in life due to Bottleium ownership. Information regarding Bottleium ownership was asked by the questions, “How was maintaining the tank?” and “Did you have any problems?” The information regarding fish ownership was asked by the questions, “How did you look after your fish?” and “Did you have any problems?” The effect of fish ownership was asked by the questions, “Did owning fish make any changes in your daily life?” and “Did you talk about your fish to others?” The interview time varied from 40 to 120 min. All conversations were recorded and transcribed afterwards.

Transcripts were analyzed using a thematic analysis method [17]. The verbatim transcripts of the 19 participants were inductively analyzed [18]. First, the qualitative data were familiarized by reviewing the field notes, listening to the recorded interviews and reading the transcripts. Then, the first author extracted tentative themes as groups of salient phrases. The themes were discussed and definite themes were developed. A conceptual model was illustrated to show thematic relationships. The labels of themes were changed repeatedly until consensus on the labels that correctly identified the themes was reached. Moreover, in the closing stages of data collection and analysis, we ensured that no new themes were extracted [19]. To enhance the validity of the analysis, the results were discussed with interdisciplinary experts in gerontology at the University of Tokyo. A qualitative software package, NVivo 11 (QSR International Pty Ltd., Victoria), was used due to its versatility for thematic analysis. Participant observation was continued and their conversation about fish was followed for approximately one year after the workshop. The method of the follow-up is as follows. The Institute of Gerontology operates the community space where the workshop was held. This community space is usually used to host various types of events targeting older adults. Workshop participants and other older adults visit the place occasionally and causally converse with each other. Researchers frequently visited the community space for operation, about once per week, and records of conversations were made when participants talked about their Bottleium [20]. The recorded conversations were analyzed using the same thematic analysis method described above.

### 2.4. Questionnaire

The participants were asked to answer a questionnaire at the end of the workshop and at the beginning or the end of the follow-up interview. The sex and the age were asked along with the following questions of previous experience of pet ownership, and current pet ownership in the first questionnaire. Participants answered the latter two questions, “Do you have previous experience of pet ownership?” and “Do you currently own pets?” in a yes or no format. Interaction with fish, such as talking to fish (“Did you talk to your fish?”: 1. Often talked, 2. Sometimes talked, 3. Rarely talked, 4. Did not talk), along with the difficulty of maintaining and handling the Bottleium was asked with the second questionnaire. Maintaining the Bottleium was asked by a question, “How hard was maintaining the Bottleium?” The participants chose from 1. Hard, 2. Somewhat hard, 3. Somewhat easy, 4. Easy. Handling the Bottleium was asked in the similar format.

## 3. Results

### 3.1. Participant Characteristics

The participant characteristic is shown in Table 1. The average age of participants was 78.5 ± 6.7, 94.7% (*n* = 18) were females, and 78.9% (*n* = 15) of them lived alone; 84.2% (*n* = 16) had previous experience of pet ownership and 5.3% (*n* = 1) owned a pet when they joined this workshop and received a fish.

### 3.2. Handing and Maintaining the Bottleium

Maintaining, changing the water and cleaning the bottle, was not hard for 94.7% (*n* = 18) of the participants, while 5.3% (*n* = 1) answered a little bit hard. The participants had to move the Bottleium for cleaning or to avoid direct sunlight. That difficulty was asked by easiness of handling the Bottleium; 57.9% (*n* = 11) said that handling the Bottleium was easy and 31.6% (*n* = 6) answered somewhat easy.

### 3.3. The Effects of Fish Ownership

From the questionnaire survey, 73.7% (*n* = 14) of the participants reported that they named the fish and all of the participants reported that they talked to the fish. From the qualitative interviews and the observational study, we extracted five themes: “observation of fish and water snail”, “interaction between the fish and the owner”, “taking care of the fish as pet owner”, “facilitation of interpersonal interaction”, and “development of support system.” The first three themes were about the owner and the animals in the Bottleium, and the latter two themes were about the workshop participants and their personal relationships. The characteristics of each theme are shown below, and examples of participant quotes are shown in Table 2.

#### 3.3.1. Observation of Fish and Water Snail

Participants actively observed the movements and behavior of the fish and water snail. They observed the color patterns and physical features of the animals. They also closely observed animal behaviors such as foraging behavior of the fish and moving speed of water snail. Participants reported that they lost track of time when observing the fish.

#### 3.3.2. Interaction between the Fish and the Owner

Participants routinely greeted the fish; in the morning, they would say *ohayo* (good morning), and at night, *oyasumi* (good night). They also used Japanese terms of greeting that one says to his or her family when leaving home, *ittekimasu* (I’m leaving), and when coming home, *tadaima* (I’m home). They also spontaneously talked to fish. For example, they tell fish to stay down when changing water, or tap the bottle and tell the fish to come to them. The participants reported that they feel relaxed when interacting with fish.

#### 3.3.3. Taking Care of the Fish as Pet Owner

Taking care of the fish and water snail was not as burdensome as caring for mammalian pets, but they still require some care. Participants changed the water in the Bottleium and fed the fish occasionally three times a week. Many marked a calendar to keep track of feeding and water changing. The season of workshop and interview was summer: a few participants reported that they kept their air conditioner running for the fish when they left the house. One participant covered the Bottleium with a cloth to shut out the light for fish at night. The owners were being very protective. Furthermore, they prioritized fish in their lives. The participants considered that taking care of the fish was one of the ways to stay active.

#### 3.3.4. Facilitation of Interpersonal Interaction

Participants reported that ownership of Bottleium facilitated interpersonal interaction. When the participants gather, they talk about fish right away. Then, they exchanged information about the fish, water plant, and water snail and also visited each other’s houses to observe fish. Older adults who did not own the Bottleium also visited their friend’s house for this purpose.

When building the Bottleium, a fish and a water snail were added to the bottle at the end. Some participants’ water snail laid eggs and babies were born, while the water snail of other participants did not. The water snail has to be removed from the bottle since they continue to rapidly reproduce. However, some were reluctant to remove baby snails. Therefore, participants with many baby water snails started to give their water snail to other participants without baby snails. In addition, as another example of a support, one of the participants supported others by buying a water plant and giving it to other participants who wanted to replace them.

Furthermore, the Bottleium facilitated interaction between family members in participants with children or spouse. A daughter called the participant since she was worried about fish, and increase in conversation was reported between married couples. In terms of conversation, triggering and spreading of conversation was observed during the observational research. For example, a participant asked a friend passing by to come in to the community center and take a look at her Bottleium. The others took interest in that interaction and also joined to look. This led to group conversation.

#### 3.3.5. Development of Support System

Two cases were reported where participants got hospitalized. Prior to going to hospital, they asked their friends or other participants to look after their Bottleium. Participants shared their worries and made promises to look after each other’s Bottleium in times of hardships.

## 4. Discussion

**Figure 2:**
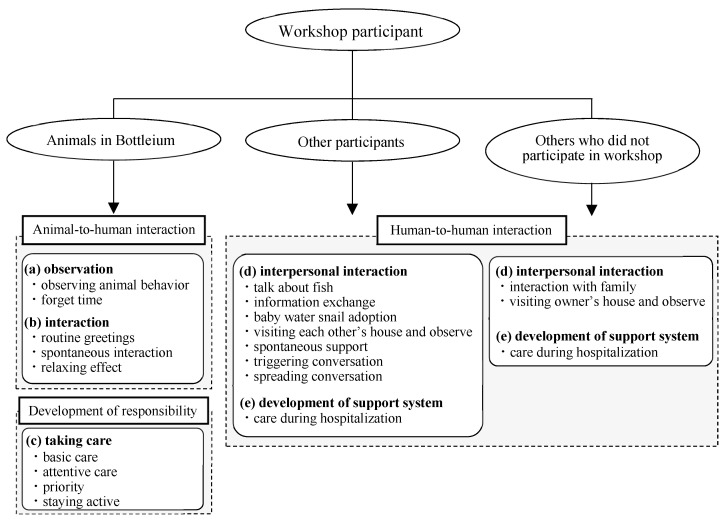
The relationship between workshop participant and animals in Bottleium, other participants and others who did not participate in workshop.

The results from the qualitative interviews and observational study implied that Bottleium ownership acted as a positive stimulus to the lives of community-dwelling older adults. The relationship between workshop participant and animals in Bottleium, other participants, and others who did not participate in the workshop, is shown in Figure 2. Themes (a) observation and (b) interaction represent animal-to-human interaction, (c) taking care suggests development of responsibility, and (d) interpersonal interaction and (e) development of support system imply the promotion of human-to-human interaction.

The promotion of animal-to-human and human-to-human interaction was observed in this study. Having a pet-like character in the living environment approaches the psychological well-being of older adults: for example, Raina et al. reported that dogs may substitute for human companionship [7]. Furthermore, the ownership of an animal-like robot “*paro*” was associated with decreased depressive moods in the geriatric adults [21]. The human-to-human interaction is equivalent to being socially engaged, and previous research has reported the benefits of social inclusion [22,23,24], particularly that the higher the number of social connections, the lower the risk of depressive mood. These studies imply that having a partner or personal interactions and actually conversing or communicating contribute to the psychological well-being of older adults. The ownership of a Bottleium provided opportunities for both animal-to-human and human-to-human interactions, which might eventually lead to maintaining or improving their psychological state.

The theme “development of responsibility” might be specific to owning a living organism. Having a pet or living animal that may die without the owner’s care could attribute to the development of responsibility. A study by Aoki et al. in 2015 reported that having a role or responsibility is significantly related to feeling the sense of *ikigai-kan*, a purpose of life [25]. Not having responsibilities in daily life, that is, a lower feeling of *ikigai-kan*, is linked to the risk of experiencing depressive moods [24]. Ornamental fish ownership could have increased the sense of *ikigai-kan* for the participants. The burden of responsibility would have been heavy if older adults were supplied with mammals as pets, since they require more care than fish. The fish in the Bottleium required occasional feeding and water change in a week, which might have contributed to applying the adequate sense of duty to the owner.

The results of this study might relate to the character specific to the region where the study was conducted. The place is an old bed town developed in 1964, and many of the residents have lived in the place ever since. Most of the participants of the workshop also shared this characteristic and participants already were familiar with each other, if not, can easily relate to a friend of a friend. The theme “development of a support system” and a part of a theme of “interpersonal interaction”, such as visiting each other’s houses, is an example of a case that could have occurred due to this condition. Although already familiar with each other, the residents at the housing complex owned the same pet for the first time through this intervention. The increase in the frequency of conversing could have led to deeper relationships among owners, and owners and non-owners. In addition, some previous Bottleium workshops hosted before this study gathered participants from different regions. After the workshop, participants dispersed back to their own towns. If workshops can be hosted at places where people are already familiar with each other, such as commuter towns or nursing homes, similar results could be anticipated.

The owners of the Bottleium did not feel difficulty in maintaining or handling the Bottleium. Older adults might be reluctant to welcome a new pet or install culturing equipment due to their age and decline in physical function. For example, Participant H said during the interview that compared to a regular aquarium that requires frequent large-scale washing, the Bottleium requires less care and that condition could be suitable for older adults. This suggest that the features of the Bottleium, for example, the size and the ease of maintenance of a system without a filter or aeration system, could be preferred by older adults. The specific product, Bottleium, was used in this study, but the term *bottleium* is also an all-inclusive term for small bottle aquariums with the same features. Similar results could be seen if easy-to-handle culturing conditions are recreated using other materials.

This study has some limitations. First, the life span of ornamental fish used in this study is approximately two years. The observational research in this study only covers the preliminary effect of pet ownership with the Bottleium. Looking after the fish can become a routine, and the death of fish could also affect older adults negatively, and future research should be continued to study this. Second, most of the older adults who gave their consent to participate were females. The report from the Japanese Cabinet Office shows that the percentage of male older adults who participate in hobby activities is low compared to female older adults [26]. This tendency could associate with low participation of male older adults. A method to involve male older adults in the follow-up research should be considered. For example, hosting a workshop dedicated to male older adults or asking female participants to invite male older adults could improve male recruitment. Having a high number of female participants could have led to the extraction of themes such as interpersonal relationships. However, many males were also involved in the conversation and similar results could be seen if male participants are recruited.

## 5. Conclusions

Overall, this study elucidated that the effect of ornamental fish could be positively affecting the lives of community-dwelling older adults. Community-dwelling older adults living independently usually have the potential to own a pet that does not require too much care. Ornamental fish, with an easy-to-maintain system like the Bottleium, could be one of the options that suit the ability of older adults. The fish and water snail were kept at home but led to facilitation of social interaction, potentially reducing the risk of social isolation. The ornamental fish used in this study typically live for about two years. In the future, research should be conducted to elucidate the effect of its death on older adults.

## Figures and Tables

**Table 1:** Participant Characteristics (N = 19).

Characteristics	Percentage (*n*)
**Sex**	
Male	5.3% (1)
Female	94.7% (18)
**Age**	
60s	10.5% (2)
70s	42.1% (8)
80s	47.4% (9)
**Living Status**	
Alone	78.9% (15)
With spouse	21.1% (4)
**Current pet ownership**	
Yes	5.3% (1)
No	94.7% (18)
**Previous experience of pet ownership**	
Yes	84.2% (16)
No	15.8% (3)

**Table 2:** Themes and Narrative Sample from Interview and Observational Research. The first ID is the actual speaker of the narrative, and the following IDs are participants who spoke similar narrative.

From Interview			
ID	Theme	Sub-theme	Narrative example
a	**Observation of fish and water snail**		
		observing animal behavior	*“The fish spits out the flake and eat it again and spits again out. They spit out big flakes.” (Participant ID C. H)*
		forget time	*“I usually clean the room in the morning but (observed the fish and) when I realized, it was time to leave home.” (Participant ID B, ID C)*
b	**Interaction between fish and the owner**		
		routine greetings	*“I call my fish’s name and then say ‘ohayo (good morning)’ (everyday).” (Participant ID D, G, L, N, O, R)*
		spontaneous interaction	*“The fish seems to jump out when changing water. So I say ‘No, don’t come out’ (to fish).” (Participant ID A, ID G)*
		relaxing effect	*“I take care of my husband at home. So right now, it is like I have two people to take care of. But when I take care of fish, it sooths me.” (Participant ID M, O, P)*
c	**Taking care of the fish as pet owner**		
		basic care	*“I wake up in the morning and exercise, feed fish, and prepare my own meal.” (Participant ID T).*
		attentive care	*“The weather seems hot and when I leave, I feel like leaving the air conditioner on for fish (because I worry)” (Participant ID A, ID B and ID C and ID F)* *“I changed the water using Natural Water (bottled water) and the water was cleaner than usual. I am going to try it again.” (Participant ID F).*
		priority	*“(after owning Bottleium) The center of my life is fish now. Fish First.” (Participant ID A, ID B)* *“When I wake up in the morning, (the first thing) I look at fish and when I sleep, I tell it that I am going to turn the light off now. Everything is ‘fish’ now.” (Participant ID C)*
		staying active	*“I first thought that the purpose of the workshop was to build a small aquarium without fish. But at the end of the workshop, we each got a fish. At first I was a bit afraid but owning a living animal keeps me active.” (Participant ID P)*
d	**Facilitation of interpersonal interaction**		
		talk about fish	*“When we gather, we talk about fish right away.” (Participant ID C)*
		information exchange	*“I learned (from other owner) that you have to remove baby water snail (because the numbers will increase drastically), so I did” (Participant ID I)*
		baby water snail adoption	*“I adopted by snail from Participant ID B.” (Participant ID A, R).*
		visit each other’s house and observe	*“This morning, my friend came to my house and I showed my fish.” (Participant ID O)*
		supporting each other	*“Participant ID E went to buy the water plant for us. He went and gave some to other participants too.” (Participant ID B).*
		interaction with family	*“My daughter is worried about the fish and calls me frequently. She asks me if the fish is ok.” (Participant ID B).*
**From Observational Research**			
ID	Theme	Sub-theme	Narrative example
d	**Facilitation of interpersonal interaction**		
		information exchange	*“(Non-participant, Male, 80s) I hear that water snails gather on the grass.”*
		baby water snail adoption	*“My snails died so I got some more from Participant ID B” (Participant ID C, L)*
		visit each other’s house and observe	*“(My friend) comes to my house and watch the fish for two to three hours. (To other friend) Please come to watch next time!” (Participant ID B).*
		triggering conversation	*“(Non-participant, Male, 80s) When I see someone, I don’t ask but the first thing they say is ‘My fish is doing well’ (because they know that I know, about their Bottleium ownership).”*
		spreading conversation	*“(To other participant walking by) Come in! (to the community space) Please look at my Bottleium. (Participant ID T).” -responding-“Is this my granddaughter (water snail)? (Participant ID B)”-others joining-“(Non-participant, Male, 80s) There are so much babies”.*
e	**Development of support system**		
		care during hospitalization	*“The lady said that she’s going to get treated in the hospital. So I volunteered to take care of her fish” (Non-participant, Male, 80s)* *“I had two Bottleiums in my house for a while when the other lady had to go to emergency. She called me and asked me to take care of fish after calling the ambulance because she was going to the hospital.” (Participant ID B)*

## Data Availability

Not applicable.
